# Negative symptoms at different stages of schizophrenia development

**DOI:** 10.1192/j.eurpsy.2026.12154

**Published:** 2026-07-31

**Authors:** N. O. Maruta, Y. A. Kushnir, M. M. Denysenko

**Affiliations:** 1 Borderline Pathology, “Institute of Neurology, Psychiatry and Narcology named after P. V. Voloshyn of NAMS of Ukraine” SI, Kharkiv; 2 Department for providing psychiatric and palliative care № 15, Communal Non-commercial Enterprise “Clinical Facility for Mental Health Care ”PSYCHIATRY" of executive body of Kyiv City Council (Kyiv City State Administration), Kyiv; 3 Emergency Psychiatry and Narcology, “Institute of Neurology, Psychiatry and Narcology named after P. V. Voloshyn of NAMS of Ukraine” SI, Kharkiv, Ukraine

## Abstract

**Introduction:**

Negative symptoms (NS) in schizophrenia are associated with the chronicity of the pathological process, its unfavorable course, a small proportion of individuals who achieve remission and a low level of social functioning.

**Objectives:**

To study NS in schizophrenia at different stages of the pathological process (first episode of schizophrenia, exacerbation and remission).

**Methods:**

The research methods included the use of clinical and psychopathological, psychometric methods (PANSS, SANS, SAPS, AES-C, PSP) and statistical methods for processing the obtained data.

**Results:**

The study was based on the examination of 331 patients diagnosed with schizophrenia (252 patients with a predominant clinical picture of NS, 79 patients with a predominant positive symptoms). Among patients with NS, 83 were included in the first episode group, 88 patients in the exacerbation group, and 81 patients in the remission group. Typical patterns of NS dynamics in schizophrenia were identified, which reflect the sequence of development of clinical manifestations at different stages of the disease and are important for predicting the course and building individualized rehabilitation strategies. In the first episode of schizophrenia, such manifestations as emotional flattening (71.4%, DC=7.84, MI=1.17, p<0.01), decreased initiative (74.2%, DC=7.89, MI=1.18, p<0.01) and general passivity dominated, which were partially reversible, in a number of cases masked by affective disorders. In the acute stage, there was an increase in the severity of abulia (64.9%, DC=6.98, MI=1.02, p<0.01), anhedonia and speech inhibition, reactivity to external stimuli decreased, patients became less involved in interpersonal interaction. In the remission stage, generalization of motivational deficit (82.1%, DC=8.83, MI=1.25, p<0.01), prevalence of apathetic-abulic syndrome, deepening of cognitive inertness (when compared with the first episode of schizophrenia) was noted.

**Conclusions:**

It was determined that NS is not a static phenomenon, but has a certain clinical dynamic, within which individual structural components of NS change their intensity, clinical and prognostic significance.

**Disclosure of Interest:**

None Declared

